# Biochar Produced from Saudi Agriculture Waste as a Cement Additive for Improved Mechanical and Durability Properties—SWOT Analysis and Techno-Economic Assessment

**DOI:** 10.3390/ma15155345

**Published:** 2022-08-03

**Authors:** Kaffayatullah Khan, Muhammad Arif Aziz, Mukarram Zubair, Muhammad Nasir Amin

**Affiliations:** 1 Department of Civil and Environmental Engineering, College of Engineering, King Faisal University, Al-Ahsa 31982, Saudi Arabia; mgadir@kfu.edu.sa; 2 Al Bilad Bank Scholarly Chair for Food Security in Saudi Arabia, The Deanship of Scientific Research, The Vice Presidency for Graduate Studies and Scientific Research, King Faisal University, Al-Ahsa 31982, Saudi Arabia; 3 Department of Civil and Construction Engineering, College of Engineering, Imam Abdulrahman Bin Faisal University, Dammam 31451, Saudi Arabia; maaahmed@iau.edu.sa; 4 Department of Environmental Engineering, College of Engineering, Imam Abdulrahman Bin Faisal University, Dammam 31451, Saudi Arabia

**Keywords:** green concrete, biochar, compressive strength, durability properties, SWOT, techno-economic analysis

## Abstract

The Kingdom of Saudi Arabia generates an enormous amount of date palm waste, causing severe environmental concerns. Green and strong concrete is increasingly demanded due to low carbon footprints and better performance. In this research work, biochar derived from locally available agriculture waste (date palm fronds) was used as an additive to produce high-strength and durable concrete. Mechanical properties such as compressive and flexural strength were evaluated at 7, 14, and 28 days for control and all other mixes containing biochar. In addition, the durability properties of the concrete samples for the mixes were investigated by performing electric resistivity and ultra-sonic pulse velocity testing. Finally, a SWOT (strengths, weaknesses, opportunities, and threats) analysis was carried out to make strategic decisions about biochar’s use in concrete. The results demonstrated that the compressive strength of concrete increased to 28–29% with the addition of 0.75–1.5 wt% of biochar. Biochar-concrete containing 0.75 wt% of biochar showed 16% higher flexural strength than the control specimen. The high ultrasonic pulse velocity (UPV) values (>7.79 km/s) and low electrical resistivity (<22.4 kΩ-cm) of biochar-based concrete confirm that the addition of biochar resulted in high-quality concrete free from internal flaws, cracks, and better structural integrity. SWOT analysis indicated that biochar-based concrete possessed improved performance than ordinary concrete, is suitable for extreme environments, and has opportunities for circular economy and applications in various construction designs. However, cost and technical shortcomings in biochar production and biochar-concrete mix design are still challenging.

## 1. Introduction

The construction industry is one of the most rapidly growing industries globally. The substantial growth of the construction industry led to the increasing demand for concrete. The population in urban regions is likely to grow from around 3.4 billion in 2009 to 6.5 billion by 2050. It is estimated that the yearly concrete production withstood at 10,000 Mt and is expected to rise twice in the next 40 years [1]. As a result, with time, there is an increasing demand for green building materials and eco-friendly construction practices to reduce the environmental impact of the concrete industry [2,3]. Carbon dioxide (CO_2_) emission has always been a severe worldwide concern in cement manufacturing. Moreover, activities related to the processing and transportation of cement are also responsible for greenhouse gas emissions, which are considered a severe environmental threat. Concrete contributes to approximately 8% of the entire world’s production of CO_2_ during the production, processing, and preparation phase [4,5]. As a result of the high CO_2_ emissions and environmental issues, it has become necessary to implement sustainable CO_2_ reduction methods relevant to cement-based materials (CBM) in the environment [2]. Environmentally friendly cementitious materials (CBM) can entirely or partially replace cement to reduce the negative environmental impacts of concrete production [6]. In recent years, various waste materials such as fly ash, silica fume, glass, rubber and tires, steel slag, etc., have been utilised in concrete to improve performance and lower carbon footprint [7,8,9]. The use of these materials has a two-fold benefit. It reduces carbon emissions by reducing the use of cement in concrete, but it also diverts waste materials away from landfills, which helps increase sustainability.

Biowaste, including municipal, industrial, agricultural, and other forms, is widely generated worldwide. This waste can be dumped into landfills and cause severe environmental consequences [10]. Transforming waste into value-added products for various applications via various approaches is a step toward a circular economy and an effective waste management strategy [11]. The pyrolysis technique is most commonly adopted to convert various biomass sources such as organic industrial and household waste, wood, and agricultural waste into biochar, biogas, and bio-oil [12,13]. Biochar is a carbon-rich material with high porosity obtained via thermochemical conversion of biomass in the absence of oxygen [14]. Recent studies showed that a pyrolysis temperature of > 500 °C releases all the organic components from biochar leading to high surface area biochars [2,15]. The biochar’s excellent mechanical and thermal stability, high surface area, and porosity proved it to be favourable to use as a cement replacement as an admixture in concrete [16,17]. For instance, Choi et al. [18] partially replaced the cement by adding biochar in a mortar and reported replacing 5% biochar showed a 10% increase in the compressive strength. Correspondingly, substantial improvement in mechanical strength (16–20%), water penetration (40%), and water absorption (35–60%) by the addition of biochar produced from various feedstock were reported [3,19]. Furthermore, Restuccia et al. [16] stated that adding biochar to cement paste could enhance its fracture energy and modulus of rupture. Wang et al. [20] reported that biochar in concrete enhances the mechanical properties by reducing the microcracks in the concrete and improving cement’s hydration. Nevertheless, there is still a considerable gap in investigating the effect of biochar from various feedstock on concrete’s mechanical and durability properties. The Kingdom of Saudi Arabia (KSA) is one of the largest producers of date palm trees, with about 35 million trees. These trees generate large agricultural waste, either carried to landfills or burned in the open areas. This has a significant detrimental impact on humans and the ecosystem [14]. Walid et al. [21] stated an efficient and valuable use of ash obtained from date palm waste as a partial replacement of Portland cement in concrete structures. Though, the addition of biochar derived from date palm wastes for construction application has not been studied yet.

The primary aim of the presented research is to study the impact of biochar derived from date palm fronds as an additive in concrete to produce high-strength and durable concrete. The control and all other biochar-containing mixes assessed compressive and flexural strengths after 7, 14, and 28 days. Additionally, the durability properties of the concrete samples for the mixes were evaluated by measuring their electric resistivity and ultrasonic pulse velocity. Finally, the SWOT analysis and techno-economic assessment of the biochar-concrete system were performed to provide detailed insight into date palm derived biochar’s potential application on a commercial level.

## 2. Materials and Methods

### 2.1. Materials

Type-1 Ordinary Portland cement (OPC) from a Saudi cement factory was used as a primary binder in all concrete specimens [22]. The cement’s specific gravity and maximum particle size were recorded as 3.14 and 0.072 mm, respectively, provided by the local manufacturer. Biochar was produced using date palm fronds at 500 °C with a heating rate of 10 °C/min for 2 h. Industrially available sand was utilised as a finer aggregate (FA) in all concrete mixtures. A 0.49% absorption capacity and 2.6% specific gravity were recorded in FA [23]. The particle size distribution investigation showed that particles passed 100% from the ASTM sieve size #4 (2 mm), 100% from sieve size #8 (4 mm), 100% from sieve size #16 (600 µm), 74% from sieve size #30 (425 µm), 12% from sieve size #50 (212 µm), 6% from sieve size #100 (150 µm) and 5% from sieve size #200 (75 µm). Pulverised limestone was added as a coarser aggregate (CA) in all mixtures. Absorption, bulk specific gravity, and maximum particle size was recorded as 1.19%, 2.57, and 18.5 mm. Tap water is used as a mix of water in fabricating all concrete specimens.

### 2.2. Preparation of Biochar-Concrete Specimens

**Figure 1:**
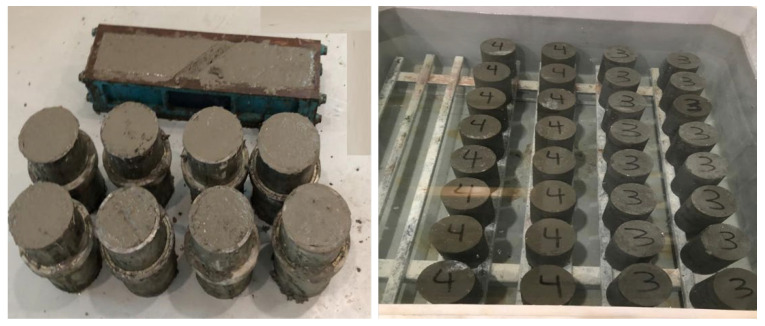
Concrete cylinder and beam specimens.

A total of 6 mix ratios were established to calculate the impact of biochar in standard strength concrete; 16 cylinders and two beams were cast for each mix. Five mixes contain various dosages of biochar, and one mix consists of ordinary Portland Cement (OPC) only. Biochar was added to the mix at a rate of 0.25%, 0.50%, 0.75%, 1.00%, and 1.50 wt% of the overall volume of concrete (Table 1), representing one mix with OPC only and five mixes with different percentages of biochar addition, as described by Akhtar et al. [2]. The water to cement ratio was kept constant at 0.45. MasterGlenium 110M was utilised as a superplasticiser. Initially, materials were dry mixed for about 2 min in a rotary mixer containing special blades. Then, water was added and mixed for another 3–4 min to achieve a homogenous mix. All specimens were cast as per ASTM C192 [24]. Beam specimens (100 mm × 100 mm × 500 mm) and cylindrical specimens (100 mm × 200 mm) were fabricated after putting the concrete mixture in the individual moulds in two consecutive layers and compacted each layer for 10 sec. Afterwards, it was levelled and covered the surface for 24 ± 2 h for setting and then submerged in a curing tank at room temperature 26 ± 2 °C for 28 days. Water curing helps to minimise the loss of mixing water from the concrete’s surface, and the additional water accelerates the strength gain. After 28 days, the samples are taken out from the curing tank to determine the mechanical properties of the concrete (Figure 1).

### 2.3. Experimental Tests on Concrete Specimens

#### 2.3.1. Compressive Strength

**Figure 2:**
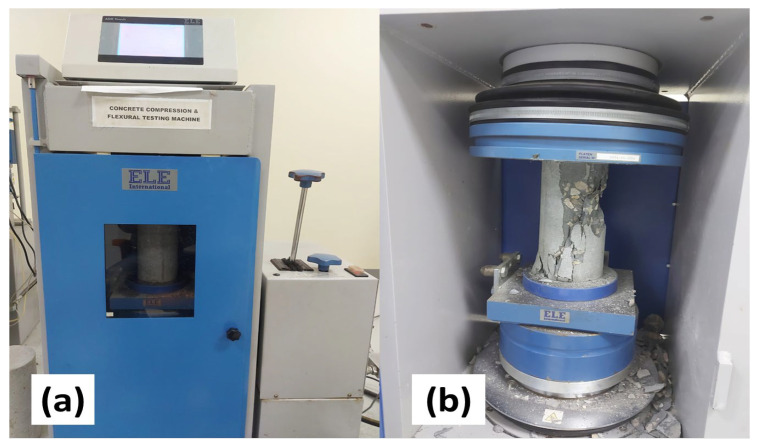
(**a**) Compressive Testing machine (**b**) Crushed cylindrical specimen.

After 7, 14, and 28 days of water curing, the compressive strength of cylindrical specimens was determined in three-time intervals. From each mix, three specimens were tested on a compression testing machine (CTM) with a loading rate of 2.4 kN/s, and the average value was noted according to [25] (Figure 2).

#### 2.3.2. Flexural Strength

**Figure 3:**
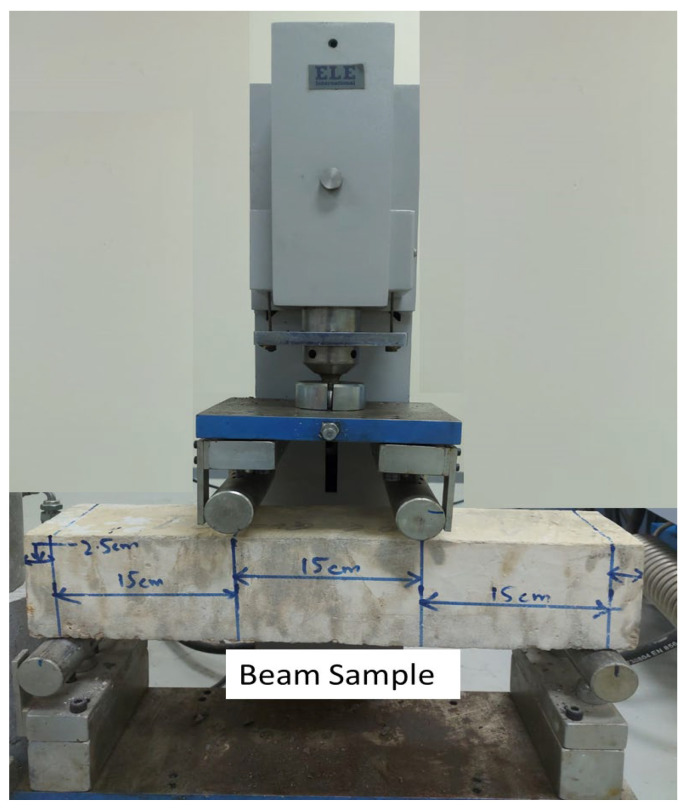
Setup for flexural testing of beam specimen.

Beams with (100 mm × 100 mm × 500 mm) were utilised to calculate the flexural strength of concrete beams after 7, 14, and 28 days of water curing. Two beam specimens from every mix were assessed on a flexural testing machine with a consistent loading rate of 0.05 kN/s, and the mean value was noted according to (ASTM C 78) [26] (Figure 3).

#### 2.3.3. Electric Resistivity

**Figure 4:**
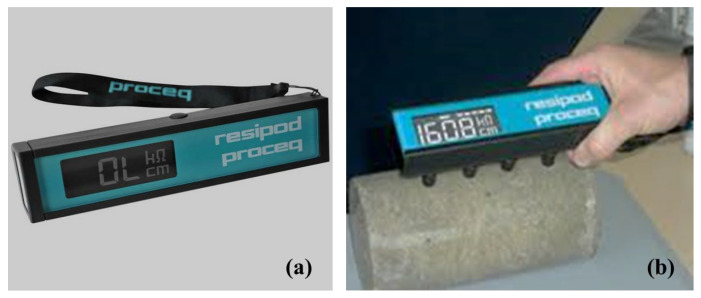
(**a**) RESIPOD electrical resistivity apparatus (**b**) Test setup to calculate electrical resistivity.

The electrical resistivity of concrete was determined using nondestructive test equipment RESIPOD as per (ASTM C1876) standard [27] (Figure 4). This test measures the bulk electrical resistivity of moulded specimens or cored segments of hardened concrete after 28 days of curing. The samples were tested after 28 days of water curing based on the practice of Su et al., 2002 [28].

#### 2.3.4. Ultrasonic Pulse Velocity

**Figure 5:**
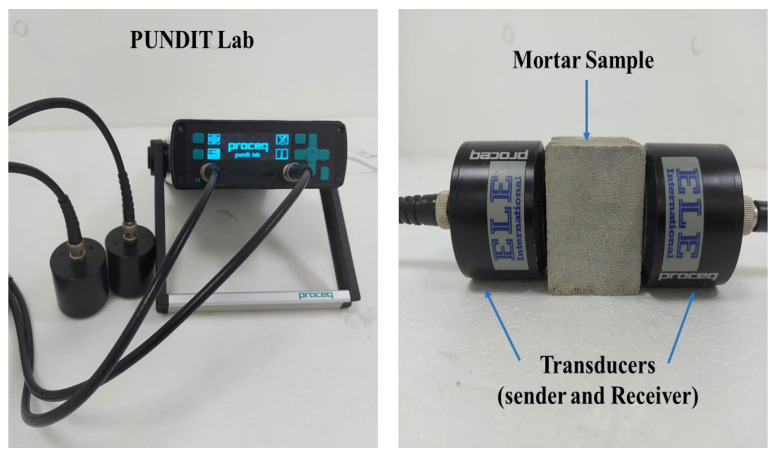
Ultrasonic pulse velocity test setup.

The ultrasound pulse velocity of concrete specimens from each mix was determined using a portable ultrasonic nondestructive digital indicating tester (PUNDIT), according to (ASTM C597) standard [29], after 28 days of curing, the same practice was adopted by Aziz et al. [30] previously. The setup of equipment for PUNDIT is shown in Figure 5.

## 3. Results and Discussion

### 3.1. Compressive Strength

**Figure 6:**
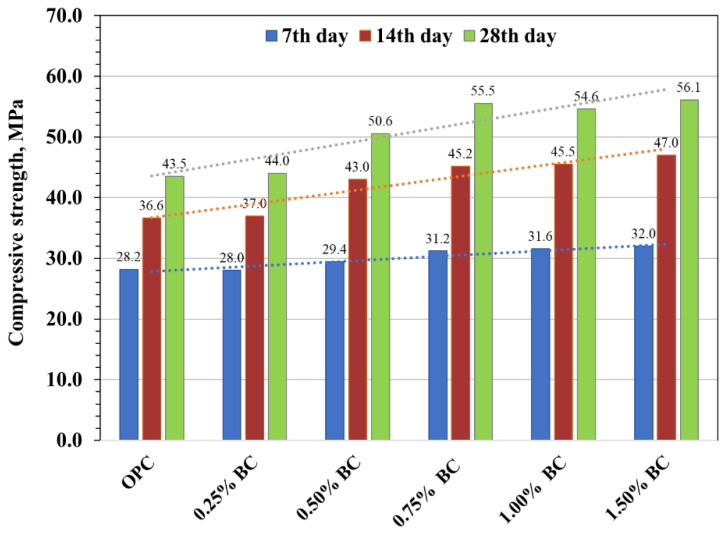
Compressive strength of concrete cylinder specimens.

Figure 6 represents the compressive strength improvement at 7, 14 and 28 days of curing cylindrical specimens made with date palm fronds biochar and compared with a biochar-free control mix. The compressive strength of the control mix was noted to be 28.2, 36.6, and 43.5 MPa at an interval of 7, 14, and 28 days, respectively. The incorporation of biochar showed a linear rise in compressive strength. For instance, adding biochar with a dosage of 0.75%, 1.00%, and 1.50% increased compressive strength by 11%, 12%, and 14%, respectively, while at 0.25% and 0.50%, biochar addition represents similar strength to control mix. A similar result on compressive strength due to biochar addition was also indicated at the 14-day and 28-day ages of concrete. It is observed that the addition of 0.50%, 0.75%, 1.00% and 1.50% of biochar indicated the strength improvement of 17%, 23%, 24% and 28%, respectively, at 14-day age and 16%, 28%, 26% and 29%, respectively, at 28-day age. However, a 0.25% biochar addition represents no significant change in strength compared to the control mix. The increase in compressive strength due to biochar’s addition was mainly associated with the biochar’s high surface area, porosity, and water retention capability [3,18]. Dry biochar particles absorbed some of the mixing water during concrete mixing, resulting in a reduced free water–cement ratio in the concrete matrix. The presence of capillary water causes the cementitious matrix to have a high capillary porosity, which has a negative impact on strength development [31]. During the initial hardening of concrete, the water absorption through porous biochar resulted in the increasing density of the cement matrix by lowering the available water in the pores. The water absorbed in the pores of the biochar was eventually provided internally to assist cement hydration via internal curing, which contributed to the cementitious matrix’s strength development [4]. Additionally, the smaller particle size of biochar exhibited a filler effect, which helps to minimise voids and gaps between cement particles and aggregates [3]. The findings discovered that the optimum biochar dosage was 0.75% and 1.50%, representing compressive strength improvement at 7, 14, and 28-day age compared to control specimens.

### 3.2. Flexural Strength

**Figure 7:**
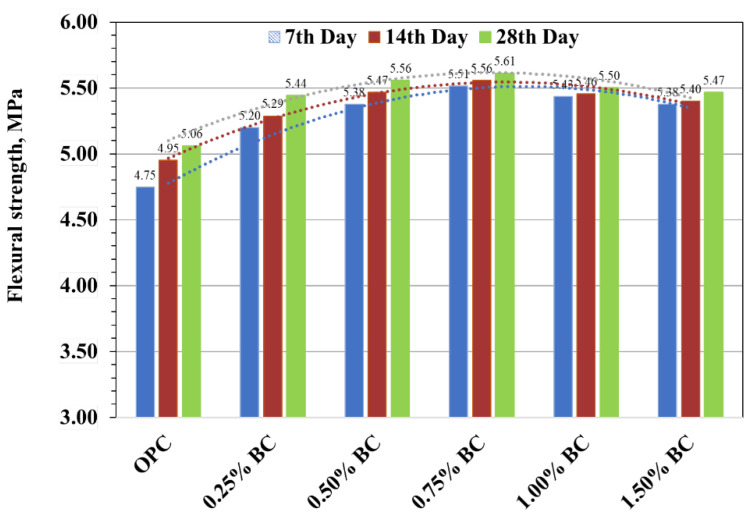
Flexural strength of concrete beam specimens.

The flexural strength biochar concrete specimens are displayed in Figure 7 at 7, 14, and 28 days of testing. The results indicate that, unlike compressive strength, adding biochar, even at minimal dosages (0.25–0.5 wt%), was favourable to enhance the flexural strength of the concrete beam. The flexural strength of the control mix was recorded as 4.75, 4.95, and 5.06 MPa at 7, 14, and 28 days, respectively. The flexural strength of all biochar-concrete mixes showed higher flexural strength than the control specimen. Adding biochar from 0.25 to 0.75 wt% in concrete showed a linear increase in flexural strength. However, with high biochar loading above 0.75 wt%, the flexural strength was not significantly enhanced. For instance, at a 7-day age, 0.25%, 0.50% and 0.75% biochar indicated a 9%, 13% and 16% improvement in flexural strength as compared to the control mix. Consequently, an increase in biochar loading, i.e., 1.00% and 1.5%, caused only 14% and 13% improvement in flexural strength, which was almost similar to 0.75% biochar-concrete but still better than the control mix. Likewise, at a 14-day and 28-day age, similar trends were noticed, 0.25%, 0.50% and 0.75% biochar resulted in around 7%, 10%, and 12% increase, respectively, at 14 days and 8%, 10%, and 11% increase, respectively, at 28 days while 1.00% and 1.50% biochar showed 10% and 9% increment at 14 days and 9% and 8% increment at 28 days in flexural strength than that of control mix. The substantial improvement in flexural strength due to the addition of biochar could be due to the flexibility provided by biochar in concrete, which functions as a link between biochar particles and hydrated cement, preventing premature fracture. Maljaee et al. [32] concluded that biochar-based mortar’s flexural strength improved due to the addition of biochar. The concrete becomes dense and tough due to the addition of porous biochar, contributing micro-reinforcement effect. This ultimately resists the crack propagation, deflects the crack path, and increases flexural strength [2]. However, a negative impact was noticed after adding biochar by greater than 0.75%. A large amount of biochar in the cement matrix may cause the aggregation of biochar particles leading to an increase in inhomogeneity in the tensile plane of the cement-biochar matrix. Similar behaviour was also reported by Ahmed et al. when using biochar in a cement composite [33]. The results indicate that the optimum biochar dosage was 0.75%, and the flexural strength produced by 0.75% biochar was improved up to 16%, 12%, and 11% at 7, 14, and 28 days compared to the control specimen.

### 3.3. Ultrasonic Pulse Velocity (UPV)

**Figure 8:**
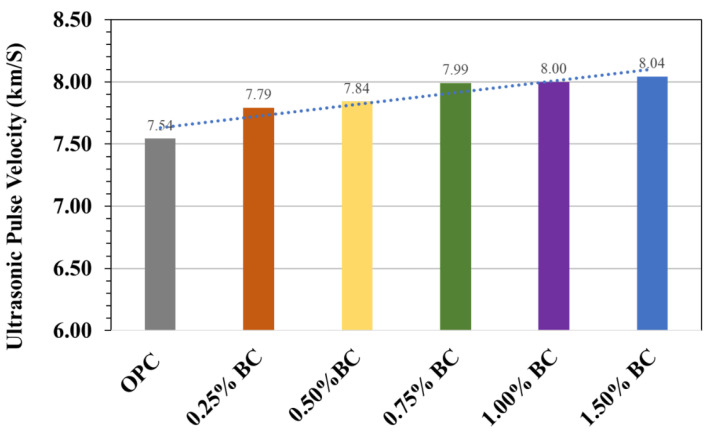
Ultrasonic pulse velocity for various concrete specimens.

In contrast to destructive testing, the research group focused on determining the mechanical properties of biochar-based concrete using non-destructive testing. Figure 8 shows the ultrasonic pulse velocity (UPV) testing results for control and biochar concrete. According to ASTM C 597 [29], the test technique has been validated and standardised. It indicates that the values of UPV were around 7.54 and 8.04 km/s, with a mean of 7.79 km/s, according to the given experimental data. However, compared to control concrete, the biochar-concrete samples had higher UPVs. The development in UPV results within biochar-concrete can be attributed to the pozzolanic activity due to the incorporation of biochar. Similar conclusions have been observed by other researchers [34,35]. It is worth mentioning that the UPV values for all the biochar-concrete samples were over 7.79 km/s, indicating that the quality of concrete is exceptional [36]. According to the literature and standard, it can be concluded that the biochar-concrete with UPV value > 7.79 km/s indicates that it is free from internal flaws, large voids, cracks, and segregation leading to reduced structural integrity and good concrete quality in terms of density, uniformity, homogeneity [37,38].

### 3.4. Electrical Resistivity ρ (kΩ-cm)

**Figure 9:**
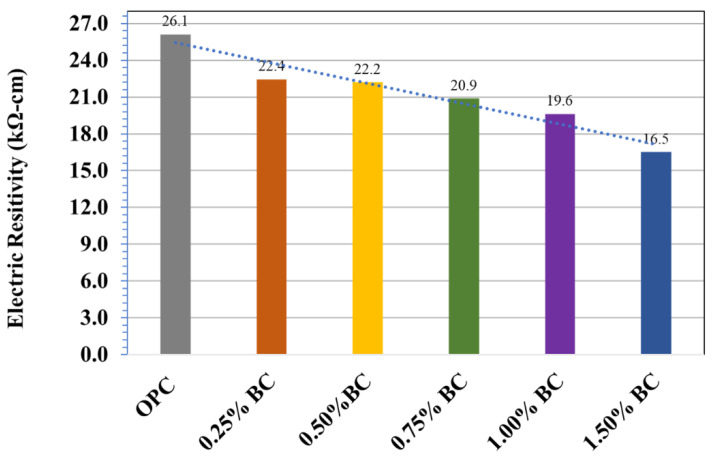
Electric resistivity of different concrete samples.

The electrical resistivity of biochar-concrete and the control specimen is displayed in Figure 9. It is indicated that the electric resistivity *ρ* decreases gradually with increased biochar content. The value of electric resistivity was recorded as 26.1 kΩ-cm for the control mix. The values of electric resistivity noticed for 0.25%0.50%,0.75%,1.00%,1.50% biochar-concrete specimen was 22.4,22.2, 20.9,19.6,16.5kΩ-cm, respectively, which indicate linear decrease in the values of electric resistivity. Any material’s electrical resistivity (*ρ*) is described as its ability to resist the ions transfer exposed to an electrical field. It mainly relies on the microstructure elements associated with the shape of interconnection and pore size [39]. A finer pore network with fewer connections results in lower permeability, leading to increased electrical resistivity [40,41,42].

### 3.5. SWOT Analysis

Concrete is considered an essential building material globally and widely used for various construction applications. However, concrete manufacturing accounts for substantial greenhouse gas emissions associated with cement production. Therefore, innovative approaches toward green concrete building materials reduce environmental and climate impact and promote sustainable societal development. Recently, biochar-based concrete gained increasing attention due to its sustainability and improved mechanical and durable properties compared to ordinary Portland concrete [2,43,44,45] However, to critically evaluate its potential for real-time application, it is necessary to summarise its merits, demerits, and limitations. Therefore, in this section, the SWOT analysis is carried out as a sustainable approach focusing on business strengths, weaknesses, profiting from opportunities, and potential identified threats of date palm derived biochar-based concrete to gain insight into and guide the relevance of the adoption of biochar in the construction industry. Table 2 summarises the main components of the SWOT analysis of date palm derived biochar-concrete construction, which is discussed below.

#### 3.5.1. Strengths

The major strengths of using date palm fronds derived biochar-based concrete as building materials are listed in Table 2. Accordingly, date palm-derived biochar-concrete possessed desirable characteristics to develop a sustainable and green concrete material without compromising its mechanical and durable properties. The date palm-derived biochar exhibits a porous graphite carbon structure and high surface area, which facilitates the formation of denser concrete, ultimately improving the compressive and flexural strength to 29% and 16%, respectively. Previous studies revealed that biochar-based concrete demonstrated comparatively better compressive strength than ordinary concrete. It was reported that the compressive strength increased to around 31% using paper sludge-derived biochar concrete after curing for 28 days [2]. In another study, adding biochar (0.08 wt% of cement) improved the compressive strength to 85 MPa and 100 MPa. Similarly, adding coarse-sized biochar (140 µm) particles to concrete may enhance the flexural strength of concrete. It was observed that 0.5 wt% of coarse biochar-based concrete, after curing for seven days, indicated a 51% higher flexural strength (3.34 MPa) than the reference concrete [46]. The date palm-derived biochar concrete demonstrated higher UPVs (7.79 km/s), indicating improved concrete durability, which is attributed to the reduction of large voids, and internal cracks in the concrete matrix. The biochar concrete exhibited a low electrical resistivity value (*ρ*), which is 47% lesser than the control concrete, suggesting that the biochar-concrete matrix consists of a heterogeneous structure with a strongly connected pore network.

Additionally, studies also confirmed that the high thermal stability of biochar-based cement composites is another essential factor demonstrating its applicability compared to ordinary concrete. It was reported that biochar-based mortar specimens consisting of different proportions (5%, 10%, and 20% of cement weight) when subjected to different heating environments (200 °C, 450 °C, and 700 °C), showed minimal % loss in strength compared to ordinary mortar [47]. The study reported that adding 5 wt% biochar retained nearly 88%, 76%, and 38% of compressive strength when exposed to high temperatures (200 °C, 450 °C, and 700 °C). Biochar produced at high pyrolysis temperature exhibits high thermal stability, significantly improving concrete fire resistance [40]. The fire stability characteristic attracts applications in concrete structures used in mines and tunnels, reducing human risks and substantial damage. Moreover, the highly porous structure of biochar serves as a thermal insulator in a concrete matrix. Generally, the low interfacial adhesion of biochar with cement matrix leads to poor heat transfer, leading to decreased thermal conductivity. It was reported that biochar derived from the peach shell and apricot, when added to concrete, showed low thermal conductivity of 0.40 and 0.34 × 10^−6^ m^2^/s, respectively [48,49]. Therefore, using biochar in concrete as cement replacement improves the mechanical, durable, and thermal properties of concrete and reduces the CO_2_ emissions of the concrete industry. The study evaluated the impact of various governing factors, including raw materials, methods, synthesis, and transportation of biochar-concrete systems on the environment. It was estimated that approximately 0 to 20 wt% of biochar additions might expect to reduce 0.15–0.20 kg of cement in concrete. Therefore, using low cement amounts in biochar-concrete may be expected to reduce greenhouse gas emissions, ozone depletion, climate change, and hazardous biowaste management [50].

#### 3.5.2. Weakness

While other agricultural wastes such as rice husk ash, bagasse ash, palm oil fuel ash, etc., have been widely used to replace OPC in cementitious materials, there is little knowledge and availability on the properties of biochar made from date palm fronds and cementitious materials from it in most regions of the world. As a result of this lack of understanding, date palm fronds biochar application is very limited in the construction industry due to the lack of high confidence in the material. Furthermore, the effect of biochar on composite cement performance still requires various experiments to be completed to draw a more accurate conclusion. It is imperative to conduct an imminent study that examines the properties of date palm fronds biochar and its impact on cementitious materials’ long-term performance to promote the practical application of date palm fronds.

The use of date palm fronds biochar in cementitious materials also has a weakness: its lower strength at high volume percentages. However, higher date palm fronds biochar content (1.5%) in the cementitious mix reduces its engineering performance, limiting its application as a binder component in cementitious materials [51,52]. Conversely, other agricultural wastes, such as rice husk ash, wheat straw ash, palm oil fuel ash, etc., are effective even at high dosages (up to 20%) in cementitious materials [53,54]. Combining date palm fronds biochar with high-reactive materials such as nano- and micro-silica makes it possible to use high dosages of date palm fronds biochar without affecting its engineering properties [55,56]. However, the date palm fronds biochar amount in the cementitious matrix must be carefully controlled since it can reduce free water and, consequently, the fluidity of concrete, increasing the demand for superplasticisers.

Similarly, cementitious materials that contain date palm fronds biochar have a longer setting time, making them less suitable for applications requiring shorter setting times. A chemical additive such as an accelerator can be added to cementitious materials incorporating DPFA as a replacement for OPC to shorten their initial and final set times [57].

#### 3.5.3. Opportunities

Growing sustainability awareness in the construction industry has led to the search for sustainable materials that can replace OPC in cementitious materials. Due to its chemical properties and the fact that it is derived from agricultural waste, date palm frond biochar is an excellent alternative source of revenue for developing sustainable concrete. Saudi Arabia ranks among the world’s leading date-producing countries. As a result of the high production of dates, the date palm industry produces a tremendous amount of agricultural waste. If these wastes are improperly disposed of in the environment, they could pose a fire and safety risk. In addition, valuable land spaces could be depleted, and the aesthetics of the environment might be impacted. The processing of these date palm fronds into biochar would provide an opportunity to efficiently manage these waste materials and use them as a raw material for making green concrete. Further, converting these wastes into valuable resources would entail a monetary value for date palm factories, opening up another source of revenue.

The application of date palm fronds biochar in building materials has been shown to improve the mechanical properties of building materials and enhance the durability of composites under extreme environmental conditions. The improved properties suggest that biochar-containing building materials perform equally or even better than those without [4,58,59,60,61]. Therefore, there is a massive opportunity for date palm fronds derived from biochar-based concrete to be used for various building applications for a better design life. Due to its wide range of applications and vast production, concrete has a substantial carbon footprint, contributing to 8% of the global carbon dioxide emissions [5]. Therefore, it is imperative to look for pathways for reducing emissions within the cement and concrete industry to reduce its environmental impact [4]. The use of date palm fronds-derived biochar in building materials has the potential to reduce carbon footprints and mitigate climate change [62]. The ability of biochar to sequester carbon in stable forms and capture CO_2_ directly from the atmosphere in building materials and the addition of CO_2_-saturated biochar have a vital role. Even with a lack of studies on this topic, building materials that contain date palm fronds derived from biochar have superb possibilities for reducing carbon footprints and mitigating climate change [63].

#### 3.5.4. Threats

Various potential barriers that may restrict date palm derived biochar-concrete commercialisation of biochar-concrete are listed in Table 2. Although biochar-concrete knowledge is progressively expanding, biochar production’s cost and engineering shortcoming is still challenging. Biochar production is an energy-intensive process that may increase biochar cost compared to cement. Therefore, an efficient design for biochar production is needed, which is economically feasible, improves biochar quality, and reduces net greenhouse gas emissions. Additionally, extensive research and development are required to identify sustainable and cost-efficient alternative production approaches to mitigate energy use. The new techniques would ensure biochar strength and market potential as sustainable future materials in the concrete industry.

### 3.6. Technical and Economic Feasibility of the Biochar-Concrete System

The possible emissions of GHGs to the environment because of the decay/decomposition of the biomass are avoided by the process of valorising the biomass to produce biochar. Reducing GHG emissions of CO_2_-eq./kg in the biochar life cycle using different biomass was estimated. In addition to reducing the net emission of GHG, the use of biochar in concrete has played a vital role in improving its chemical and mechanical properties. Most studies in the past showed significant improvements in concrete’s compressive and flexural strength [3,64,65,66,67,68]. Other mechanical properties of concrete, such as toughness, flexibility, elongation, permeability, thermal stability, and thermal conductivity, were also observed [19,64,69,70]. Despite a relatively high cost of production of biochar [71], compared to natural filler materials such as sand, it is still considered a better construction material due to its associated environmental benefits of reduced CO_2_-eq./kg as well as the generation of other value-added products such as syngas, bio-oil production in pyrolysis.

Apart from the environmental and other technical benefits of using biochar in concrete, its economic viability is crucial in deploying it in the construction industry. Kung et al. [72] reported a higher and more feasible feedstock value of 10.98 $/t for biochar production by slow pyrolysis compared to a correspondingly lower value of 2.85 $/t pyrolysis. A lesser value of biochar production in fast pyrolysis is due to higher net losses of feedstock and is considered unviable both in economic and environmental profits. A more excellent feedstock value in slow pyrolysis is a viable solution for biochar production. As reported by several researchers, [73] biochar production from forest residue using a portable system, the cost of biochar production can be further reduced, equaling 470 $/t of oven-dried by technologically improving the portable system.

## 4. Conclusions

In this research, biochar derived from date palm waste was used as an additive to concrete at the different mass compositions of 0.25 wt% to 1.5 wt%. The performance of biochar-concrete specimens derived from date palm waste was examined by the fresh concrete specimen’s representative mechanical and durability characteristics. The following conclusions can be drawn based on the outcomes:

1. The compressive strength of biochar-concrete increased with increasing biochar content and showed a maximum 28%, 26%and 29% improvement in power at 28-day age with the incorporation of 0.75%, 1.00%, and 1.50% of biochar. The biochar-concrete containing 0.75 wt% biochar loading indicated 16% higher flexural strength than the control mix. The increased surface area, small particle size, and water retention capability of porous biochar lead to a denser concrete matrix, formation of cement hydrates, and filler effect resulting in stronger concrete.
2. Biochar-concrete showed high values (>7.79 km/s) of UPV demonstrating high-performance concrete. The electrical resistivity reduced linearly with the incorporation of biochar. This confirmed the formation homogeneous and denser biochar-concrete network resulting in lower permeable concrete.
3. The SWOT and techno-economic assessment analysis further corroborates that the biochar-concrete system possessed the high potential to be commercially adopted as green and sustainable material despite the economic and engineering challenges.
4. In general, it is suggested that biochar derived from Saudi agriculture waste can be used as a beneficial product for infrastructure designs requiring high-performance and durable building materials to attain technical and environmental benefits.

## Figures and Tables

**Table 1:** Mix design composition of Biochar-concrete specimen.

Specimen	Cement (kg/m^3^)	Sand (kg/m^3^)	Gravel (kg/m^3^)	Biochar (kg/m^3^)	w/c Ratio
Control	466.56	685	934	-	0.45
0.25 wt% BC	465.39	685	934	1.166	0.45
0.50 wt% BC	466.22	685	934	2.33	0.45
0.75 wt% BC	463.06	685	934	3.49	0.45
1.00 wt% BC	461.89	685	934	4.66	0.45
1.50 wt% BC	459.56	685	934	6.99	0.45

**Table 2:** SWOT analysis of biochar-based concrete.

Strengths	Weaknesses
Stronger and rigid concrete Dense concrete matrix Internal curing agent Reduce carbon footprint Reduce hydration rate High-quality concrete Low electrical resistivity	Biochar production is an energy-intensive process Biochar dispersion in concrete is not homogeneous Biochar possessed varied surface morphology Limited research High biochar production cost Lower acceptability
**Opportunities**	**Threats**
Effective use of biowaste Other products formation Biochar composite industries Biochar insulation materials Carbon sequestering material Global climate change	High energy consumption in biochar production Limited technology advancements

## Data Availability

The data used in this research has been appropriately cited and reported in the main text.
